# Editorial: Reviews in gut-brain axis: stress, dysregulation in gut-brain axis function and stress related disorders

**DOI:** 10.3389/fnins.2025.1665282

**Published:** 2025-09-03

**Authors:** Zuleide Maria Ignácio, Jonathan B. Clayton, Margarete Dulce Bagatini, Avinash Veerappa

**Affiliations:** ^1^Federal University of Fronteira Sul, Graduate Program in Biomedical Sciences, Chapecó, SC, Brazil; ^2^University of Nebraska Omaha, Omaha, NE, United States; ^3^Nebraska Food for Health Center, Lincoln, NE, United States; ^4^University of Nebraska-Lincoln, Lincoln, NE, United States; ^5^University of Nebraska Medical Center, Omaha, NE, United States; ^6^Department of Biology, University of Nebraska at Omaha, Omaha, NE, United States

**Keywords:** gut-brain axis, stress, brain function, inflammation, gastrointestinal

Emerging evidence underscores the important role of the gut-brain axis across a spectrum of neuropsychiatric, neurological, and gastrointestinal disorders. Understanding how gut microbiota influence brain function and behavior continues to interest alike. Recognizing the complexity and interdisciplinary nature of this field, we invited studies spanning diverse methodologies from bibliometric analyses and meta-analyses to Mendelian randomization (MR) approaches and clinical trial reviews. This editorial synthesizes recent findings from these varied approaches, highlighting both their innovative contributions and the methodological challenges that persist in gut-brain axis research.

Lin et al. employed bibliometric analysis to elucidate the growing interest and current hotspots surrounding bipolar disorder (BD) and gut microbiota. Their findings emphasized microbiome diversity, inflammation, and probiotics as critical areas of focus, while noting the necessity of more robust clinical trials to validate potential interventions (Lin et al.). In parallel, Wang et al. provided a comprehensive review of depression-associated gut microbes and metabolites, illustrating clear links between altered microbiota profiles, such as increased lactobacilli, and depressive symptoms. However, they cautioned the clinical community regarding inconsistent efficacy in microbiome-targeted treatments, underscoring the need for personalized, systems-level approaches to therapy (Wang et al.).

Extending beyond mood disorders, Zhou et al. used MR to explore causal relationships between gut microbiota and cortical structures implicated in neuropsychiatric conditions. Their findings notably revealed associations between gut taxa, such as Mollicutes and Tenericutes, and orbitofrontal cortical morphology, thereby proposing a biological substrate underpinning gut-brain interactions (Zhou et al.). Similarly, Qiu et al.'s MR study robustly linked the gut microbiome, specifically the family Veillonellaceae, to epilepsy subtypes, opening new avenues for targeted microbiome interventions despite unclear mechanistic pathways (Qiu et al.).

Further emphasizing neurological implications, Guo et al. reviewed the role of gut microbiota in Parkinson's disease (PD), highlighting fecal microbiota transplantation (FMT) as a promising therapeutic strategy. They detailed how gut dysbiosis exacerbates PD pathology through mechanisms like increased intestinal permeability, α-synuclein aggregation, and neuroinflammation, while also urging the need for rigorous clinical validation to substantiate therapeutic claims (Guo et al.).

Shifting focus to functional gastrointestinal disorders (FGIDs), Shuai et al. applied meta-analysis of resting-state fMRI studies, demonstrating altered brain activities, particularly in regions such as the insula and anterior cingulate cortex, among FGID patients. These findings underscore the complex interplay between gastrointestinal symptoms and brain networks, suggesting neurological targets for potential intervention (Shuai et al.).

Jiang C. et al. explored γ-aminobutyric acid (GABA) as a gut-derived therapeutic candidate for anxiety and insomnia, highlighting its neuroactive potential and advocating for engineered probiotics to enhance therapeutic efficacy. Nonetheless, they acknowledged significant gaps in validating clinical safety and effectiveness (Jiang C. et al.).

In reviewing chronic pain, Ho et al. elucidated how the brain-gut axis, mediated through microbiome dysbiosis and vagal dysfunction, significantly contributes to chronic pain mechanisms. Their narrative review proposed innovative therapeutic strategies including microbiome restoration and vagus nerve modulation, yet stressed the urgency for clinical trials to ascertain effectiveness and safety (Ho et al.).

Jiang M. et al. reviewed the microbiota-gut-brain axis's intricate role in anxiety disorders, detailing neuroimmune, endocrine, and neural signaling pathways implicated in anxiety pathophysiology. Despite promising preliminary findings, they pointed out considerable translational hurdles in moving microbiota-targeted therapies into clinical practice (Jiang M. et al.).

Additionally, Hayer et al. provided a systematic review and meta-analysis focusing on antibiotic-induced gut dysbiosis and its associations with cognitive, emotional, and behavioral changes in rodents. They reported significant associations between antibiotic intake and increased anxiety- and depression-like behaviors, as well as impaired spatial cognition. Although the findings indicate a potential causal relationship, the considerable heterogeneity in experimental designs and methodologies used across studies emphasizes the necessity for standardized approaches to enhance the reliability and translational potential of these findings (Hayer et al.).

Finally, Bertollo et al. concluded that there is an intricate interplay between the hypothalamus-pituitary-adrenal (HPA) axis and the gut-brain axis in the pathophysiology of depression. Dysregulation of the HPA axis, triggered by chronic stress, leads to elevated cortisol levels and neuronal damage in brain regions involved in mood regulation. Simultaneously, alterations in gut microbiota composition can impair gut-brain communication, promote systemic inflammation, and compromise serotonin production—factors closely linked to depressive symptoms. These interconnected pathways underscore the multifactorial nature of depression and suggest the potential of integrated therapeutic strategies targeting both neuroendocrine and microbiota-related mechanisms (Bertollo et al.).

Collectively, these studies represent groundbreaking efforts toward unraveling the complexities of the gut-brain axis across various disorders. Nevertheless, the heterogeneity of findings, coupled with methodological challenges such as inconsistent approaches, limited causal evidence, and translation gaps, highlight the necessity for integrated, interdisciplinary research frameworks. Future studies leveraging multi-omics platforms, bioinformatics, and artificial intelligence will be crucial in advancing this rapidly evolving field toward robust clinical application.

